# Experimental Investigation of Sand Subjected to High Stress Levels in Wet and Dry Conditions

**DOI:** 10.3390/ma15196775

**Published:** 2022-09-29

**Authors:** Shadi Youssef, Abdallah Accary, Christophe Dano, Yann Malecot

**Affiliations:** 1 INRAE, Aix Marseille Université, RECOVER, 13182 Aix-en-Provence, France; 2 Université Grenoble Alpes, CNRS, Grenoble INP, 3SR, 38000 Grenoble, France

**Keywords:** sand, quasi-oedometric test, experimental method, high passive confinement

## Abstract

This work aimed to understand the mechanical behavior of siliceous and calcareous sand materials under uniaxial confined compression loading at high stress levels. For this purpose, a series of quasi-oedometric compression tests were conducted on sand materials, to examine the effects of grain size, nature, and moisture contents on the soil crushability and the compression behavior, using an upgraded thick pressure vessel device that can reach mean stress up to 500 MPa. All samples were prepared using an aspect ratio of 1:1 (diameter: height), placed inside a high strength steel vessel, and compressed at a uniform axial displacement rate of 5 µm/s. The vessel is instrumented with multiple strain gauges allowing for the characterization of the hydrostatic and deviatoric behavior of each test. The results of quasi-oedometric tests, conducted on these types of sand, up to a passive confinement of 500 MPa, show that particle breakage is enhanced by the presence of water. It was noticed that, for siliceous sand, smaller particles break more than larger particles, and that the calcareous grains manifest a rapid response to axial stress compared to siliceous sand. Testing various soil properties shows a great potential to better characterize the sensitivity to breakage of soils. Lastly, a post-mortem analysis of samples before and after testing, using the X-ray micro-tomography technique, was applied to study the mechanical damage of sand specimens.

## 1. Introduction

Understanding the mechanical behavior of sand when subjected to high stress levels is one of the important topics deeply investigated in the last decades [1]. During an impact on soil, an underground explosion, a percussive drilling process, or an earthquake, a large quantity of energy is released, subjecting the soil to stress levels up to several hundreds of MPa [2]. The mechanical characteristics of soils subjected to impact loading play important roles in engineering practice, such as the dynamic consolidation method and blasting compaction. However, changes in the original soil structure under impact loading may lead to extremely complicated soil mechanical behavior [3].

The Quasi-Oedometric Compression (QOC) test has been widely used to determine the mechanical response of granular material [4]. In all previous studies, particle crushing is observed; hence, it can be said that the particle crushing is one of the most characteristic phenomena of particulate materials under high pressures.

Many experimental studies have already examined the effect of initial void ratio [5], particle size [6,7,8] and angularity [9,10,11], relative density [12,13], material composition [14,15], and saturation degree [16,17,18] on particle crushing and thus on soils’ behavior [19]. The studies of the behavior of sand media at low to intermediate levels of stress are plentiful, but are limited for compression tests at high levels of stress (some tens to some hundreds of MPa). Most of the experimental results for oedometric tests found in the literature reveal that only the axial stress–strain measurements were done [20,21]; however in this study, the radial strain along with radial stress were measured within the soil specimen based on processing of the records of strain gages. 

In addition, previous experimental results show that a one-dimensional compression curve can be generalized as existing of two phases divided by a yield point. In the first stage, the soil skeleton undergoes an elastic compression and grain rearrangement through particle sliding and rolling. In the second phase, the intergranular stresses exceed the crushing strength of the grain material causing the soil compressibility to increase drastically. Hereafter, to the best of the author’s knowledge, few results are available regarding the influence of grain size, grain nature, and the moisture content on the mechanical behavior of sand material under high stress levels (up to 500 MPa). Furthermore, it was found that the deformation and crushability behavior of the soil is highly influenced by the mineralogical constitution, particle size, as well as the initial grading and saturation state of the soil specimen. According to Gudehus [22], the relation between the stiffness of the grain and the isotropic compression was combined under a single term called “solid hardness”. The solid hardness is different from the hardness of a single grain, and it has been modeled by Bauer [23] to express the variation of both the compressibility and the void ratio with respect to the isotropic compression in dry/wet conditions.

This paper aimed to assess the effect of grain size, nature, and moisture content on the mechanical behavior of sand materials i.e., siliceous and calcareous under stress levels of up to several hundreds of MPa. First, the quasi-oedometric test device along with the upgrade of the test setup is presented. Second, the principle of the test and gauge calibration by means of numerical simulation is explained. In the third part, LVDT calibration and experimental setup validation, using a 1000 kN hydraulic press machine called the ‘Schenck Press’, available at the 3SR laboratory, on siliceous sand, is shown. Finally, quasi-oedometric test results on siliceous and calcareous sand with different moisture contents are discussed. A comparison between results obtained experimentally and provided by the image analysis of post-compacted sand specimens sorted out by X-ray tomography scans is discussed.

## 2. Static Quasi-Oedometric Test

### 2.1. Test Principle

**Figure 1:**
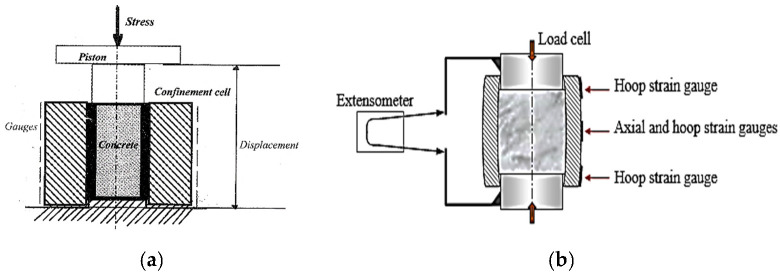
Principal of the Quasi-Oedometric Compression test: (**a**) Developed by Burlion et al. [24] instrumented with LVDT; (**b**) Developed by Forquin et al. [25], instrumented with strain gauges and an extensometer.

Static Quasi-Oedometric Compression (QOC) tests haven been widely used to get a better understanding of the uniaxial behavior of geomaterials (i.e., soil, concrete, rock) under high passive confining pressures. During this test, a cylindrical specimen is usually embedded in an instrumented steel confinement ring, and then compressed between two cylindrical plugs, as shown in Figure 1a [24]. The apparatus used herein was developed by Forquin et al. [25] to test concrete, rocks or PMMA samples for which the axial displacement measured is very small (few millimeters); the cell was instrumented with circumferential strain gauges so that the lateral stresses on the specimen could be measured during the test (Figure 1b).

### 2.2. Setup Description and Plug Modification

**Figure 2:**
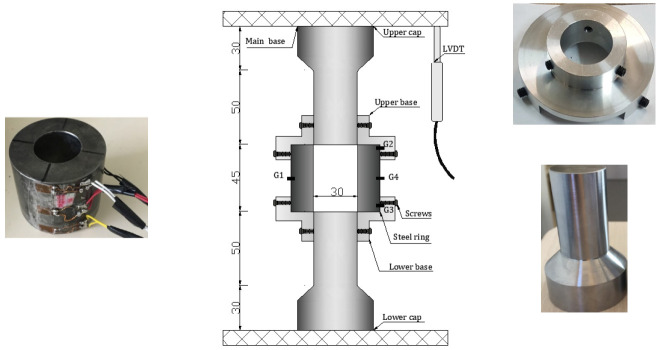
QOC test apparatus, modified set up. Steel confining ring instrumented with four gages, steel plugs and aluminum base being machined. All dimensions are in millimeters.

In the present study, a cylindrical specimen of sand material (diameter: 30 mm, length: 30 mm) was enclosed in an instrumented steel confinement ring (inner diameter: 30 mm, outer diameter: 60 mm, length: 45 mm, elastic limit about 1800 MPa) and subjected to axial compression through the testing machine. During the test on sand materials, the settlement was expected to be much higher than concrete or rock materials; however, the existing upper and lower plugs were very short, leading to an incomplete test. To circumvent this issue, the setup has been upgraded as shown in Figure 2. Two new plugs were fabricated in a way to cross the cell from side to side (diameter: 30 mm, length: 50 mm). A gap, about 0.1 mm thick, was left between the plug diameter and the inner diameter of the ring which is sufficient enough to avoid the sticking of the plug inside the ring during the test. The plugs were made of Toolox44 steel material, having the following elastic characteristics: Young’s modulus equal to 210 GPa, Poisson ratio equal to 0.3, and elastic limit equal to 1.4 GPa. In addition, to avoid any misalignment and instability of the setup at the beginning of each test, it was proposed to fabricate two aluminum bases equipped with six screws that were used to host the cell from one side and the plug from the other side (Figure 2). To ensure a good functioning of the QOC setup the maximum axial force applied was capped to 350 KN (around 500 MPa of axial stress), and the maximum pressure that can be produced between the plug and the inner wall of the ring is equal to 1 GPa. The axial stress in the specimen was calculated from the load cell. Two LVDTs were attached to the compression platens to measure the axial displacement allowing for the calculation of the axial strain. The radial stress and strain were deduced from the hoop micro-strain gauges attached to the confinement ring as follows: two gauges G1 and G4, diametrically opposite gauges at the middle of the cell, and two gauges G2 and G3 placed at ±3H/8 from the mid-plane of the ring, which are used to evaluate the barreling of the ring.

### 2.3. Strain Gages Signals Processing

**Figure 3:**
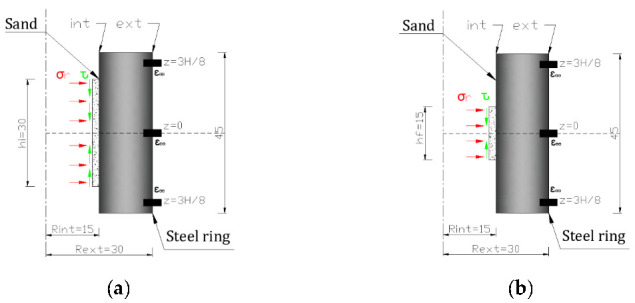
Loading definition: internal pressure and shear stress applied on the inner surface of the ring: (**a**) hi = 30 mm; (**b**) hf = 15 mm.

**Figure 4:**
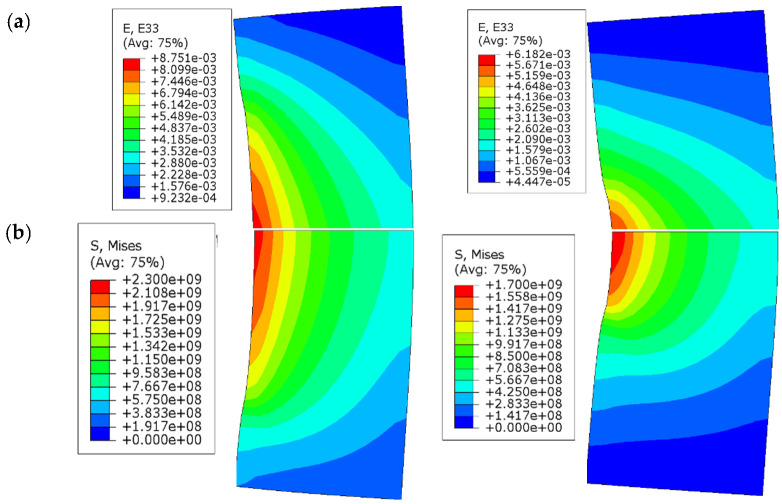
Axisymmetric numerical simulations of the elastic ring loaded as described in Figure 3: (**a**) Hoop strain; (**b**) Von Mises criterion.

The same technique and data processing methods used in previous studies [26,27,28,29] were implemented herein to compute the radial stress and strain levels, by means of the four hoop strain gauges, within the sand specimen. Axisymmetric numerical simulations using Abaqus [30] were performed by exerting an internal pressure equal to 1 GPa and a shear stress equal to 100 MPa (friction ʋ set equal to 0.1 [28]) on the inner surface of the confining ring. This latter was assumed to behave in the elastic domain (Young modulus: 210 MPa, Poisson ratio: 0.3). The pressure and shear stresses were then applied over two main heights as shown in Figure 3: hi = 30 mm that corresponds to the initial height of the sand specimen, and hf = 15 mm that corresponds to the minimum height of the sand specimen that was measured after each test. The hoop strain obtained, and the field of von Mises are shown in Figure 4a,b, respectively, for the two values of h (30 mm and 15 mm). In both cases, the maximum von Mises stress reached 2.3 GPa, which corresponds to a maximum internal pressure equal to 1 GPa and is associated with a maximum hoop strain on the external surface of the ring of 0.26%, in which the ring remains in the elastic domain.

Equations (1) and (2) [25,27,28] present the evolution of radial stress as a function of the external hoop strain at the symmetry plan (*z* = 0):

(1) $${\bar{\sigma }}_{radial}\left(h=30\right)=f_{30}\left(\varepsilon_{\theta \theta }^{z=0,ext}\right),$$

(2) $${\bar{\sigma }}_{radial}\left(h=15\right)=f_{15}\left(\varepsilon_{\theta \theta }^{z=0,ext}\right),$$

**Figure 5:**
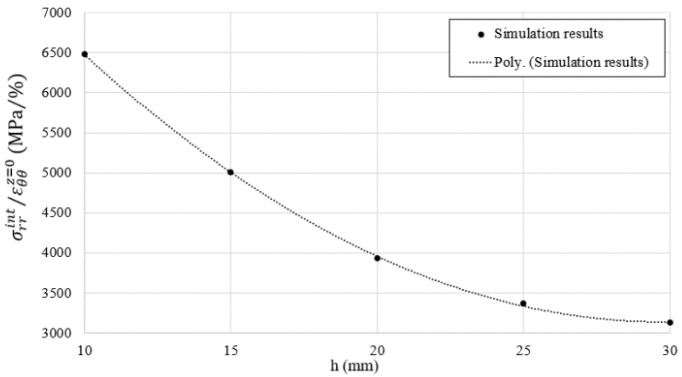
Numerical simulation results of 1 GPa internal pressure applied at five different heights. Ratio of the internal radial stress to the external hoop strain at *z* = 0.

To generalize the above equations, a series of numerical simulations have been performed on the ring (1 GPa as internal pressure and 100 Mpa as shear stress) for different heights, h, between 30 mm and 15 mm. Figure 5 presents the result of the ratio between the radial stress over the external hoop strain at *z* = 0 as a function of the loading height h. The internal pressure was then evaluated according to Equation (3) [25,27,28], where $f_{\sigma }\left(h\right)$ is a polynomial function of degree two that allows fitting the numerical data in the considered range of *h*.

(3) $$\frac{\sigma_{r}}{\varepsilon_{\theta \theta }^{z=0}}=f_{\sigma }\left(h\right),$$

Similarly, the same numerical simulations were used to evaluate the internal hoop strains ($\varepsilon_{\theta \theta }^{z=0,int},\;\varepsilon_{\theta \theta }^{z=H/2,int}$) as a function of the external hoop strains ($\varepsilon_{\theta \theta }^{z=0,ext},\;\varepsilon_{\theta \theta }^{z=3H/8,ext}$) using two coefficients of proportionality ($\alpha_{0}^{0},\;\alpha_{H/2}^{3H/8}$) for the same loading conditions applied on the same range of height (30 mm and 15 mm). After that, the average radial strain was estimated as written in Equation (4) [25,27,28].

(4) $${\bar{\varepsilon }}_{radial}=\left({\bar{\varepsilon }}_{axial},\;\alpha_{0}^{0}\varepsilon_{\theta \theta }^{z=0,ext},\;\alpha_{H/2}^{3H/8}\varepsilon_{\theta \theta }^{z=3H/8,ext}\right),$$

Thus, with only the data of the axial force, the axial strain measured using LVDT, and four external hoop strains measured by the gages attached to the vessel, one can obtain the axial stress, the evolution of the deviatoric stress, the passive hydrostatic confinement, and the volumetric behavior of the sand specimen (Equations (5)–(8) [25,27,28]). It is worth noting that all mentioned coefficients and strain values that stem from numerical simulations were updated after each test depending on the final height reached by the sand specimen.

(5) $${\bar{\sigma }}_{axial}=\frac{F_{axial}}{{\bar{A}}_{0}\;{\left(1+{\bar{\varepsilon }}_{radial}\right)}^{2}},$$

(6) $${\bar{\sigma }}_{deviatoric}=|{\bar{\sigma }}_{axial}-{\bar{\sigma }}_{radial}|,$$

(7) $$P_{hydrostatic}=-\frac{1}{3}\left({\bar{\sigma }}_{axial}+2{\bar{\sigma }}_{radial}\right),$$

(8) $${\bar{\varepsilon }}_{volumetric}=\left(1+{\bar{\varepsilon }}_{axial}\right)+{\left(1+{\bar{\varepsilon }}_{radial}\right)}^{2}-1,$$

### 2.4. LVDT Calibration

In this study, the axial displacement of the sample was only measured by means of two perfectly tuned LVDTs instrumented on the Schenck Press. Both LVDTs allowed measuring the displacement of the setup placed between the press caps, which comprised the axial displacement of the plugs and the sand together. Thus, to minimize the effect of the steel plug displacement on the sample deformation, a calibration test was recommended to subtract the axial elastic displacement of the two steel plugs from the total vertical displacement recorded by the LVDTs during the test. Three displacements were then defined:

1. $U_{\left(LVDT\right)test}$: Axial displacement registered by the LVDTs during a normal test on sand specimen.
2. $U_{\left(LVDT\right)cal}$: Axial displacement measured by the LVDTs during the calibration test.
3. $U_{sand}$: Axial displacement of the sand specimen only during the test.

**Figure 6:**
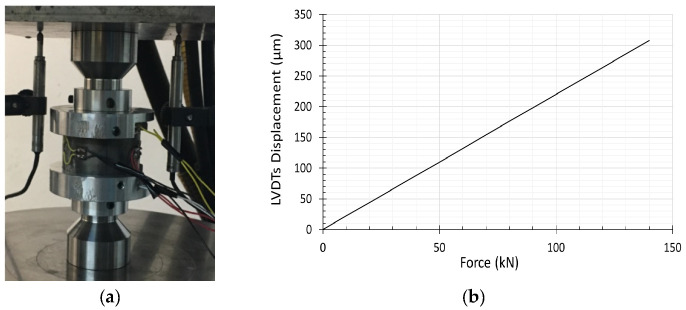
LVDT calibration test: (**a**) Test set up: the confining ring is empty; the two plugs are set at mid distance from the cell; (**b**) Test result: LVDT’s axial displacement versus the applied axial force.

Figure 6a shows the calibration test concept where the steel plugs are set at the mid distance of the ring, then compressed against each other using the hydraulic press machine to derive a relation between the steel’s displacement and the LVDT’s.

(9) $$Force=KU_{\left(LVDT\right)calibrated},$$

(10) $$U_{\left(sand\right)}=U_{\left(LVDT\right)test}-Force/K$$

Figure 6b depicts the result of the calibration test; the linear variation of the $U_{\left(LVDT\right)calibrated}$ with respect to the *Force* F indicates the elastic behavior of the steel plug material, characterized by a stiffness *K* equal to 2.14 kN/μm (Equation (9)). Finally, the displacement of the sand specimen was estimated using Equation (10). It is worth mentioning that the axial force does not affect the hoop strain placed on the confinement ring due to the gap between the latter and the steel plugs.

### 2.5. Setup Validation on Sand Material

**Figure 7:**
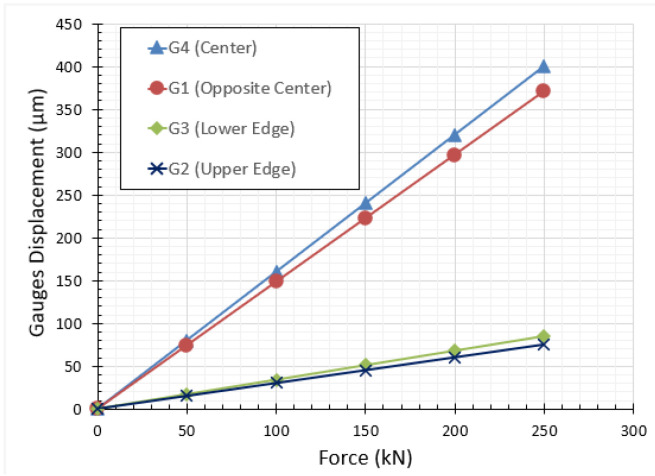
Steel ring calibration test; strain displacements were recorded by four gauges, fixed on the outer wall of the steel vessel (see Figure 2b), as a function of the applied vertical force.

**Figure 8:**
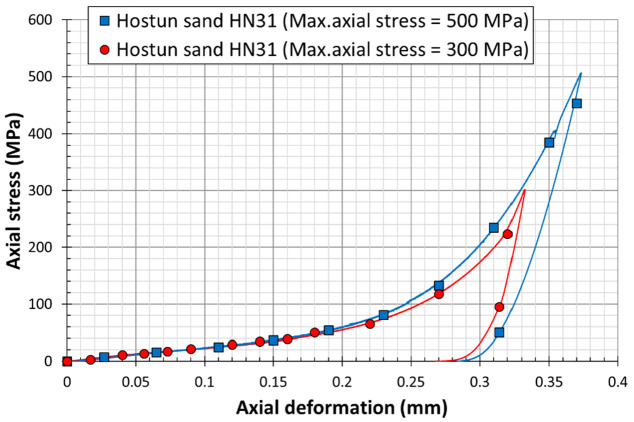
Quasi-oedometric test results conducted on Hostun sand grains (HN31) up to high axial stress levels of 500 MPa (bleu square dots) and 300 MPa (red circular dots).

Two preliminary oedometric tests were carried out on two identical specimens of a siliceous sand (Hostun sand HN31) to validate the process. Figure 7 depicts the variation of the hoop strain of all gauges with respect to the axial force applied on the specimen. The plots confirm the compatibility of the recorded strains by the two central gauges (G1, G4) and the edge gauges (G2, G3). The curves show also that the hoop strains at the center of the cell is much higher than the one at the edge due to barreling effect. Figure 8 shows the results of two quasi-oedometric tests conducted on the same material but for different values of axial force. The plots depict the variation of the axial stress on the sand specimen with respect to the axial deformation. It is clearly observed that the mechanical behavior of the sand material is typically identical for both samples; however, the sample under higher axial stress reaches higher axial deformation (red curve). After the setup validation, we considered that the proposed coefficients (Equation (4)) determined using numerical simulations, as well as the gauges’ and LVDTs recording, were valid and could be used in the analysis of sand behavior under one-dimensional confined compression.

## 3. Material Description, Test Preparation, and Validation

**Figure 9:**
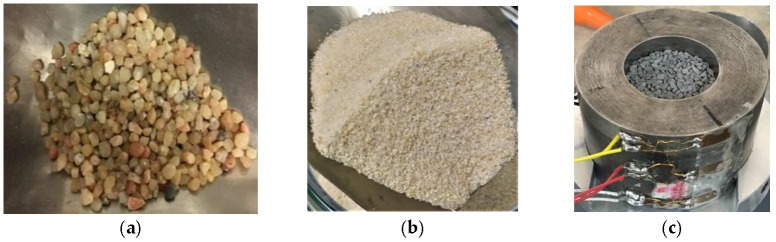
Different types of Granular material tested: (**a**) Silica sand HN1.25/1.6 (*D*_50_ = 1.415 mm); (**b**) Silica sand HN31 (*D*_50_ = 328 µm); (**c**) Limestone Glageon sand (*D*_50_ = 1.425 mm).

The main purpose of this study was to investigate the effect of particle size, grain mineralogy, and moisture content on the mechanical response when subjected to uniaxial confined compression at very high stress levels. To achieve this purpose, three granular sands were tested, namely a siliceous fine sand Hostun (HN31), a siliceous coarser sand Hostun (HN1.25/1.6), and a dry calcareous sand Glageon (GLAG1.25/1.6), derived from a carbonate rock crushed in Bocahut quarry, France, as illustrated in Figure 9. The two Hostun sands have different mean grain sizes: $D_{50}$ = 1.415 mm and 328 μm for HN1.25/1.6 (Figure 9a) and HN31 (Figure 9b) respectively. Table 1 portrays material properties. Although the three types of soil have a uniform grain size distribution, Glageon sand is composed of elongated particles compared to Hostun sand (Figure 9c).

Sand specimens were also tested at controlled moisture contents of 0%, 5%, and 10%. The characteristics of the quasi-oedometric tests conducted on sands are detailed in Table 2. Tests are referred as (HN or GLAG)-(S or L)-(0, 5 or 10), where “HN” is Hostun sand, “GLAG” is Glageon sand, S or L refers to the mean grain size (S for smaller grain size, L for larger grain size), and 0, 5 or 10 are the percentages of the water mass fraction in the sand. The sand was first oven-dried at 100 °C for at least four hours, and then mixed to ensure a consistent particle size distribution. Each specimen contained approximately 32.5 g of dry sand, to which ~1.5 g or ~3 g of water was added for a moisture content of 5% or 10% respectively. To ensure an even distribution of water, the sand was mixed in a metal pan by the usage of a glass rod.

**Figure 10:**
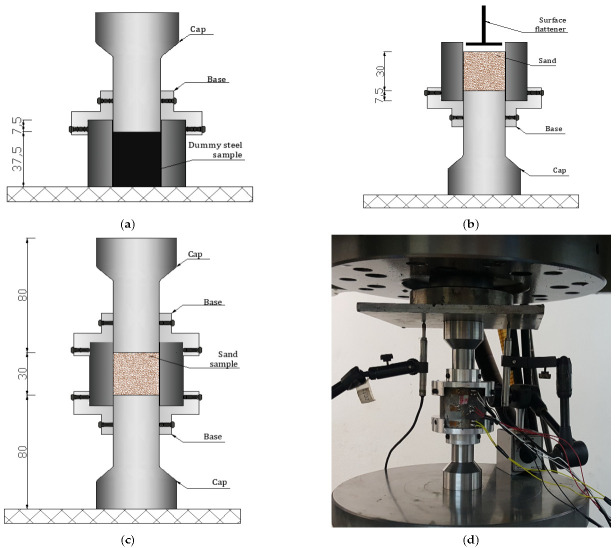
Sample preparation: (**a**) Sample height definition by means of dummy steel sample; (**b**) Pouring sand materials inside the confinement ring; (**c**) Final configuration; (**d**) Setup ready for test.

The cell has a predefined height equal to 45 mm and inner diameter equal to 30 mm as stated before. The sand sample height was set equal to 30 mm (slenderness ratio = 1), which means that the sample must be placed at equal distance from the top and bottom to ensure an accurate reading from the circumferential gages. To do so, an adequate experimental procedure has been developed following the mounting procedure below:

1. The confinement cell is placed on a steel flat support, and a dummy steel sample with 37.5 mm height is slipped inside it.
2. The steel plug is introduced inside the cell from the top, then fixed at a distance equal to 7.5 mm by means of a base equipped with six bolts (Figure 10a).
3. The whole set is inversely rotated, and both the flat support and the dummy sample are removed from the top.
4. The cell is filled by 30 mm of sand material in three layers using a funnel and small spoon. Each layer of 10 mm height is compacted using a tamper. This step will also reduce the effect of initial density on the mechanical response (Figure 10b).
5. Finally, the upper plug is slipped inside the cell for the remaining height (=7.5 mm), and then the upper base is installed and screwed to guarantee the stability of the system (Figure 10c).

Before each test, the steel cell internal wall surface was oiled to facilitate the extraction of the deformed granular specimen once the experiment is finished. The assembly was then placed below the hydraulic jack of the press. The two LVDTs (25-mm maximum axial displacement each), along with the four circumferential strain gauges (already glued on the external wall of the vessel) were connected to the acquisition system of the press machine as shown in Figure 10d.

**Figure 11:**
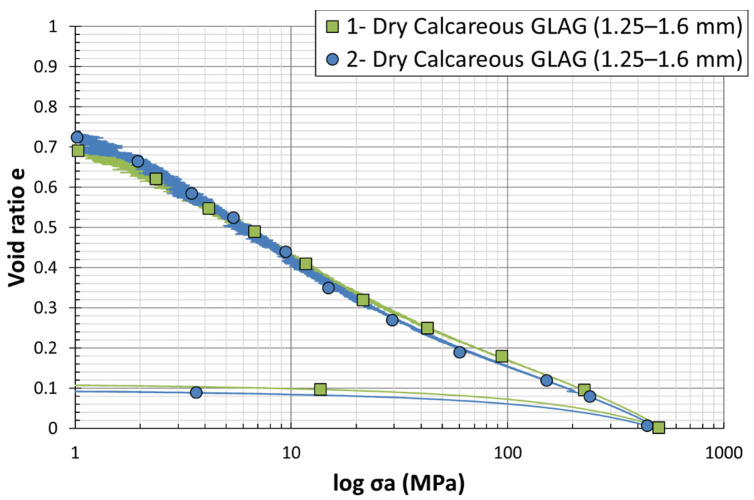
Load–settlement curves from dry oedometer tests on Calcareous sand grains of similar sizes (*D*_50_ = 1.425 mm).

Even though the good level of signals repeatability can be visualized during the oedometric test on Hostun sand materials (see Figure 7 and Figure 8), ensuring the reliability of this analysis requires a validation on the Glageon sand too. Therefore, quasi-oedometric tests were performed on two dry calcareous grains of similar sizes $D_{50}$ = 1.425 mm denoted by GLAG-L-0#1 and GLAG-L-0#2, respectively. The sample and test characteristics are given in Table 2. Figure 11 depicts the evolution of the void ratio with respect to the axial stress. The decrease in the void ratio while increasing the axial stress is almost similar for both tests; the plots follow the same curvatures and trajectory throughout the experiments. The required repeatability and reproducibility needed to validate the experimental procedure, which can be observed in Table 2, have been ensured.

## 4. Quasi-Oedometric Test Results on Sand

This section will present the experimental results of a series of dry and wet one-dimensional compression tests performed on calcareous and siliceous sands. The one-dimensional compression was regulated by the amount of axial stress applied. The later stress was taken off from zero until 400 MPa, and then the granular specimen was unloaded by 10 MPa and the axial stress decreased from 400 to 390 MPa. This was done to identify the reversible parts of the sand’s behavior (i.e., to dissociate elastic/plastic strain, evolution of elastic modulus). Again, the sample was loaded until 500 MPa of axial stress to be finally unloaded directly from 500 MPa to zero. Table 3 shows a summary of the oedometric tests results performed on the different sand materials. In Section 4.1, Section 4.2, and Section 4.3, the evolution of the void ratio, yield point, and particle breakage, associated with the effect of particle size distribution, grain nature, and water content, on the sand grains’ response are respectively discussed.

### 4.1. Effect of Particle Size Distribution

**Figure 12:**
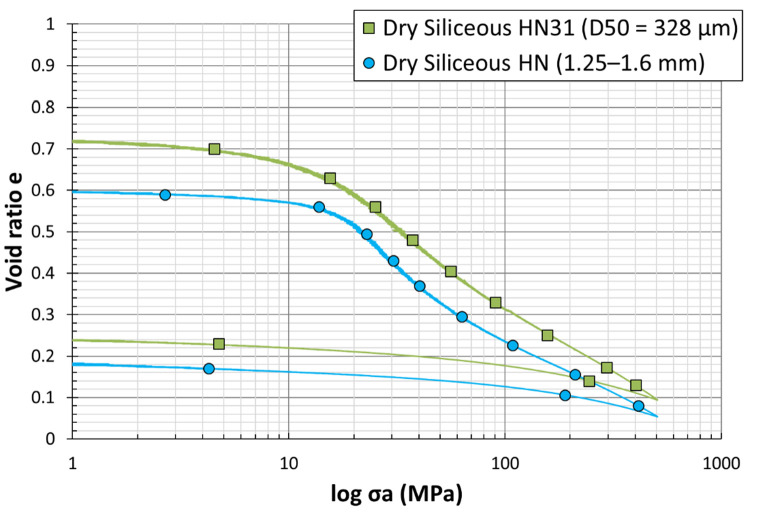
Load–settlement curves from dry odometer tests on siliceous sand grains of two different sizes (*D*_50_ = 328 µm vs. 1.415 mm).

**Figure 13:**
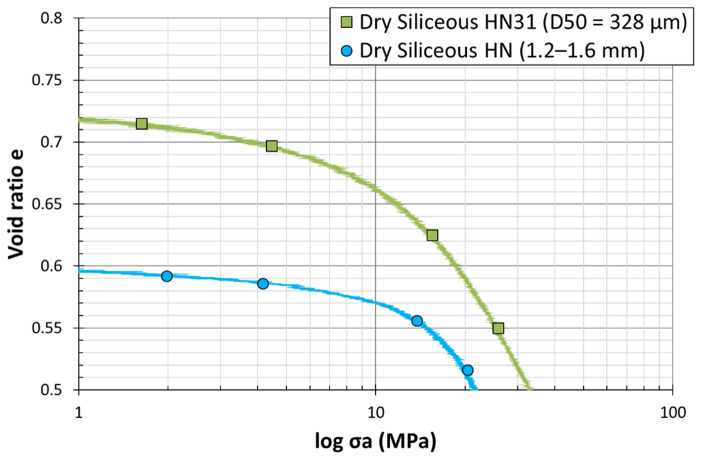
Magnification of the first part of the load–settlement curves from dry odometer tests on siliceous sand grains of two different sizes (*D*_50_ = 328 µm vs 1.415 mm). The first part represents low stress levels (σ = 100 MPa) until reaching the yield point.

Figure 12 shows the $e-log\sigma_{a}$ curve for two different grain size distributions of silica sand. At low stresses (0 to 100 MPa), the plots show a quasi-elastic behavior for both grading (Figure 13). A slight decrease of the void ratio with respect to the logarithmic vertical stress can be observed, until reaching the first inflection point, the point of maximum curvature which was defined by McDowell [4] to be the yielding point, indicating an acceleration of grain crushing (cataclastic breakage). Immediately after this point, the increase of axial stress results in a steep decrease in the void ratio, observed through an approximately linear normal compression line beyond the yield. Lee and Coop [31] deduced that particle crushing is behind the existence of normal compression, and attributed yield to the onset of particle crushing. The yield stress is known as the minimum axial stress for the initiation of particle breakage. Similarly, Huang et al. [32] introduced yield stress as critical stress and the linear slope as the compressibility index $C_{c}$ of granular materials. The previous literature on one-dimensional compression tests on samples of silica sand has shown that smaller particles reach the yield point at stresses higher than large particles, i.e., the yield stress decreases as particle size increases [5]. Therefore, the strength of a single particle increases as particle size decreases, which is not the case in the current study; as seen in the curves, the yield stress of the large particles is higher than the small ones, with a more obvious change in the slope. The plots clearly disclose a specific yield point for each sand. Considering this evidence, particle crushing enhances the compressibility of sand grains. In other words, the yield point is the best evidence for the beginning of the particles’ crushing, and the increase of compressibility index, which happened because of the increase in the vertical stress, reveals the increase in the stiffness of the specimen. The graph also shows that, when the load increases, particle breakage becomes more difficult, and this hardening subsequently leads to a stabilized particle size distribution. Based on $e-log\sigma_{v}$ plots, it is obvious that the sample expanded a little during the unloading part of the test, explained by the increase of the void ratios with the vertical stress. This is a quasi-straight line showing an exponential relationship, as described by swelling index $C_{s}\;$ [33].

### 4.2. Effect of Grain Nature

**Figure 14:**
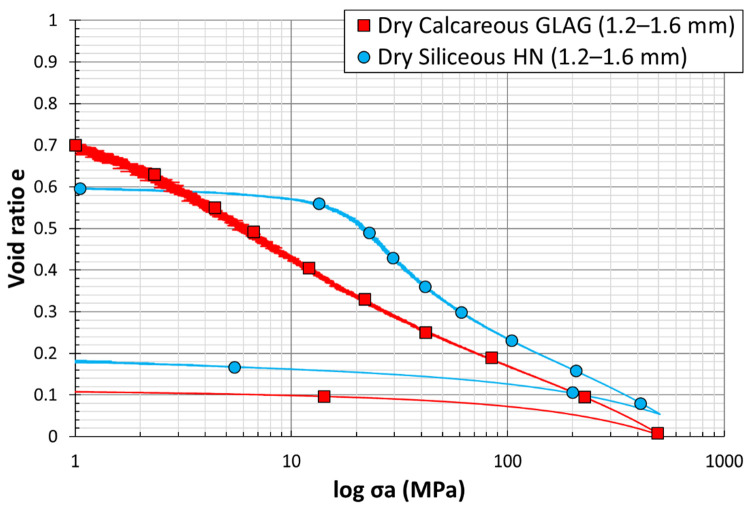
Load–settlement curves from dry odometer tests on siliceous and calcareous sand grains of same size (1.25–1.6 mm).

Figure 14 shows the variation of the void ratio e with respect to the logarithmic vertical stress $\;\sigma_{a}$, for uniaxial compression tests on dry large, calcareous Glageon and siliceous Hostun sand specimens, defined by the red and the blue plots, respectively. The mechanical response of the large siliceous Hostun grains (1.25/1.6 mm), shows a quasi-elastic behavior, with a rearrangement of the grains and a constant void ratio at low stress levels (σ = 10 MPa) until reaching the yield point. However, the mechanical behavior of the large calcareous Glageon grains (1.25/1.6 mm) does not show any elasticity. The curve revealed the appearance of the yield point at irrelevant axial stress levels (σ = 1 MPa). This observation clarifies the high crushability of GLAG with respect to HN grains. Wils et al. [17] stated that the calcareous sand, defined as crushable sand, breaks more easily compared to the siliceous grains. This difference in the behavior of both sands has been attributed to the angular shape of GLAG particles and their weaker mineralogical constitution. Suescun-Florez et al. [5] reported that silica sand shows a considerable grain breakage beyond 10% strain, which barely depends on the compression rate. However, calcareous sand discloses a totally different behavior as the particles start to split much earlier (5% of strain), with a high compression rate dependency. Note that in the current work, the strain needed for silica and limestone grain breakage was reached at almost 28 MPa and 0.84 MPa of axial stress, respectively. These stresses describe the yielding point of each specific granular material. In addition, Shahnazari and Rezvani [15], evaluated the effect of parameters that affect two different types of calcareous sands (C1 & C2), and announced that the threshold stress of the later grains is modest (C1: Bushehr Port—0.65 MPa, C2: Hormuz Island—1 MPa). The red curve—GLAG1.25/1.6—indicates that the deformation is mostly plastic; the elastic deformation is only about 2% of the total settlement. The angular shape, edges, corners, and the neighboring state of the calcareous particles make them appropriate to break under uniaxial stress. The response of these grains shows a mild slope throughout the uniaxial compression until the end of the test, when the void ratio is almost zero. In contrast, as mentioned in Section 4.1 regarding the behavior of siliceous sand, a sudden breakage occurred at the level of the siliceous grains immediately after the yield point, expressed by a steep slope with large rise over run void ratio to effective stress change. The slope starts to be slighter (around 80 MPa of axial stress) due to the presence of small new particles, derived from the splitting larger ones, in the pre-existing voids. According to Mesri and Vardhanabhuti [34], calcareous grains show more deformation and crushability at intermediate levels of stress than siliceous grains, which at low stress levels show insignificant crushing and yielding.

The void ratio then continues to decrease, i.e., indicating an increase in the compressibility index, affecting the extent of the axial stress ($e_{final}\sim 0.06)$. These observations may be referred to as the internal friction between granular particles that may change from grain type to another which depends on the nature and morphological characteristics of the grains [35].

### 4.3. Effect of Water Saturation Degree

**Figure 15:**
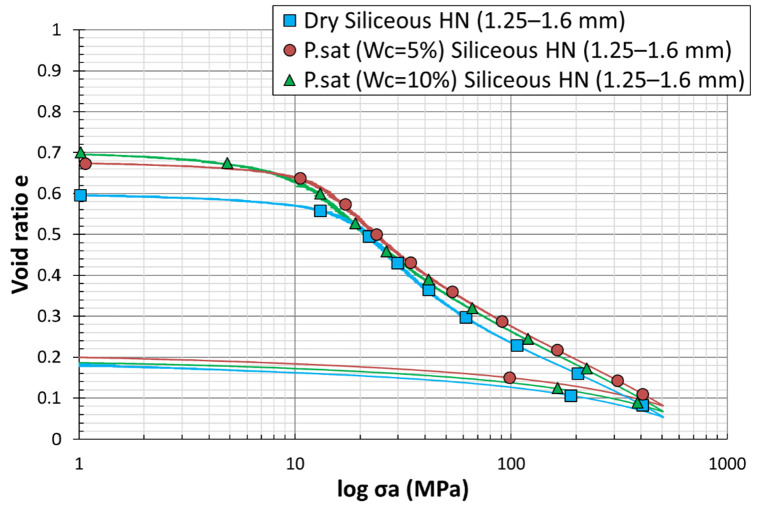
Load–settlement curves from dry and partially saturated oedometer tests on siliceous sand grains of 1.25–1.6 mm.

**Figure 16:**
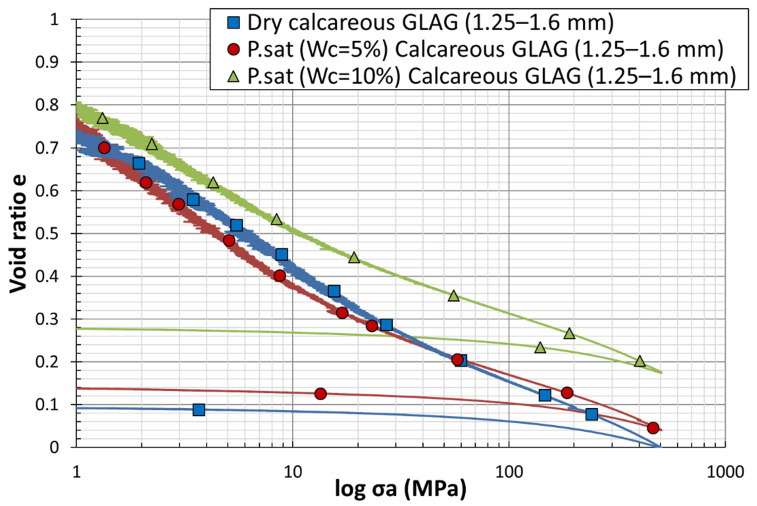
Load–settlement curves from dry and partially saturated oedometer tests on calcareous sand grains of 1.25–1.6 mm.

Figure 15 and Figure 16 present the effect of moisture content on the mechanical behavior of siliceous and calcareous sands respectively. Blue, red, and green plots in each graph refer to the one-dimensional compression tests performed on sand grains at, respectively, 0%, 5%, and 10% of moisture contents. For Hostun HN1.25/1.6 sand, the blue curve, describing the response of the dry samples, shows the higher stress, whereas the green one, referred to the partially saturated samples with $\;w_{c}=10\%$, possesses the closest yield point to the y−axis. In conclusion, the yield stress decreases as the saturation ratio increases; hence, the grain breakage is enhanced by the presence of water. Bauer [35] showed that the increase in the saturation ratio decreases the solid hardness and therefore increases the evaluation of grain breakage. The yield point of the red plot, which belongs to the compression test of partially saturated samples with $\;w_{c}=5\%$, emphasized this conclusion, as it exists between the two previously mentioned yield points. For GLAG1.25/1.6 sand, the effect of moisture content on the one-dimensional compression is almost impossible to be identified at the early stage, since the three plots show no difference, whereas Wang et al. [36] discovered that the bearing capacity of the saturated calcareous grains is 50% less than that of the dry ones. The graph demonstrates that the slopes of the normal compression line, defined before as $\;C_{c}$, of the partially saturated specimens are quite similar and gentler than the normal compression line’s slope of the dry specimens (Figure 16).

Besides, the calcareous samples show the same behavior, as the compressibility index increases with the diminution of the water content (Figure 16). Thus, the compressibility of soil decreases with increasing moisture content. Luo et al. [37] set forth that $C_{c}$ decreases with the increase of moisture content. The low moisture content used in this experimental study indicates that the initial saturations are low as well, and the specimens will not become fully saturated during oedometric tests. The specimens were locked down in the steel vessel in drained conditions, as evidenced by droplets of water leaking from the bottom side of the system during the compression. Barr et al. [38] pointed out through his experiments that dry specimens reach lower densities than wet ones for a given stress. Admittedly, the addition of water for sand specimens during quasi-static compression tests reduces grains’ stiffness. His quasi-static tests on the sub-rounded and sub-angular particles at moisture contents of 0%, 2.5%, and 5.0%, indicate that the higher densities are achieved by the wet specimens for a given stress. He also mentioned that with an approximate dry density of $2.35\;\mathrm{Mg}/m^{3}$ and a water content of 5%, the specimen can reach the fully saturated state at the end of the test. Martin et al. [39] has bound the stiffness reduction to the lubricating effect of water molecules between sand grains. The authors believe that this effect decreases the amount of friction between the particles and intensifies the sliding and rolling mechanisms, which allow them to move easily during the compression. At the final stage, once the stress reaches the maximum value (500 MPa), siliceous samples barely show a difference in the final density and thus in the void ratios. However, for calcareous samples, Barr’s interpretation is proven, since the partially saturated simples ($w_{c}=10\%$), represented by the green plot, show a higher density and the dry samples, represented by the bleu plot, illustrate a lower one. This analysis fits into the difference of the swelling index, described by the slope of the unloading part, between the plots. Even though the unloading shows slightly different behavior, the swelling index increases with the decrease of moisture content.

## 5. Post-Mortem Analysis X-ray Tomography

**Figure 17:**
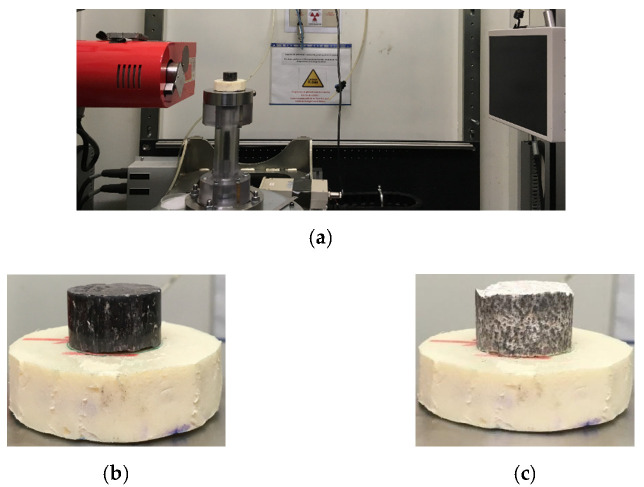
X-ray tomographic system for geotechnical studies: (**a**) The device at Grenoble has a maximum resolution of 5 µm; (**b**,**c**) calcareous and siliceous sand samples respectively, after compression.

The influence of initial void ratio, grain shape, and particle arrangement on the onset of collapse of two sand specimens (HN 1.25/1.6 and GLAG1.25/1.6) was investigated at the microscale level using the X-ray computed tomography (CT). The latter is a non-destructive imaging technique that enables the characterization of a material’s structure in 3D and allow study of the in situ behavior of geomaterials such as concrete [40], sandstone [41], and mortar [42]. The device is installed at the 3SR laboratory and allows scanning objects whose diameters vary from 4 mm to 200 mm with resolutions of 5 and 100 μm, respectively. It then becomes possible to observe the structure of the scanned object since it provides a set of images with voxel values representing the local density of the material. The X-ray parameters needed to be chosen for the scans, and they were the same for all the scans. In this paper, the range of the source tension was chosen to be equal to 150 KeV, which is considered as an optimum value for sand specimen as per Watanabe et al. [43]. The intensity was equal to 200 µA, allowing a range of different sample sizes and densities to be imaged. The time for one projection was estimated to be equal to 2.664 s/projection, so an entire scan required about one hour with a total number of images equal to 1120 per scan. The sample to be scanned was placed between the X-ray source beam and the detector on a translation and rotation stage (Figure 17a). The source and detector can be translated in the two directions normal to the axis of the beam, which allows specimens of different heights to be conceived and scanned [44]. The laboratory protocol herein consists of following the collapse of the HN 1.25/1.6 and GLAG1.25/1.6 sand specimens under the dry condition W_C_ = 0% before and after being one-dimensionally compressed (see Figure 9a,b that shows that state of material before compression). The axial and passive confinement pressure experienced by each sand types were the same and are equal to 500 and 145 MPa, respectively, as reported in Table 3. Figure 17b,c show the final state of the HN and GLAG specimens extracted very carefully from the pressure vessel to avoid extra damage.

**Figure 18:**
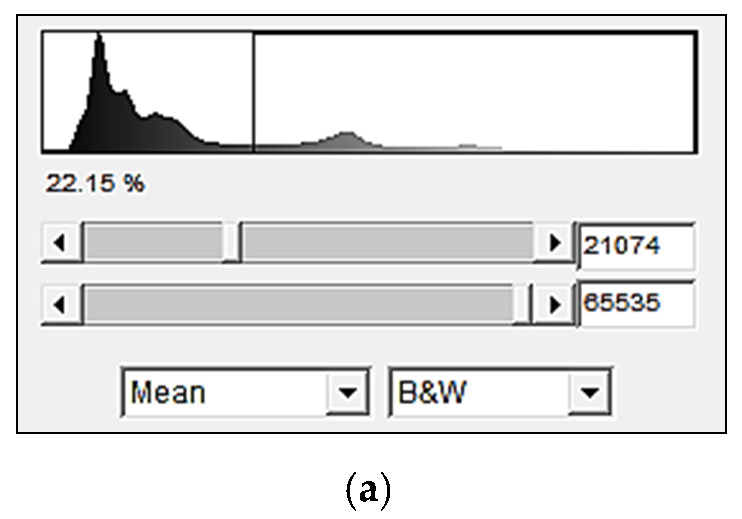

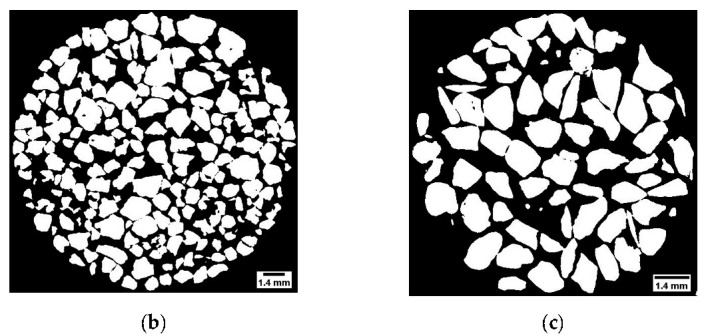
Image processing: (**a**) Grey-level histogram; (**b**,**c**) Tomographic cross-sectional view of HN1.25/1.6 and GLAG1.25/1.6 at their initial condition (before test).

The reconstruction was carried out through the so-called Fiji software, which provides registration and segmentation image processing algorithms. The threshold was done by the average method, and the 21.074 threshold has been used as the grey level to separate pixels into two classes (Figure 18a); those less than this grey-level threshold were assigned a zero value (black pixels) to represent the void volume, while all others were assigned a 255 value (white pixels) to represent the volume of solids [40]. The initial particle arrangements and shapes are shown in Figure 18b for HN1.25/1.6 and Figure 18c for GLAG1.25/1.6. The HN scan shows a sub-rounded grain shape whereas the GLAG scan shows an angular grain shape; this finding confirms the morphology of each sample as described in Table 2. In addition, it is obvious that the initial void ratio of GLAG sand is less than the HN sand sample since the latter is much denser.

**Figure 19:**
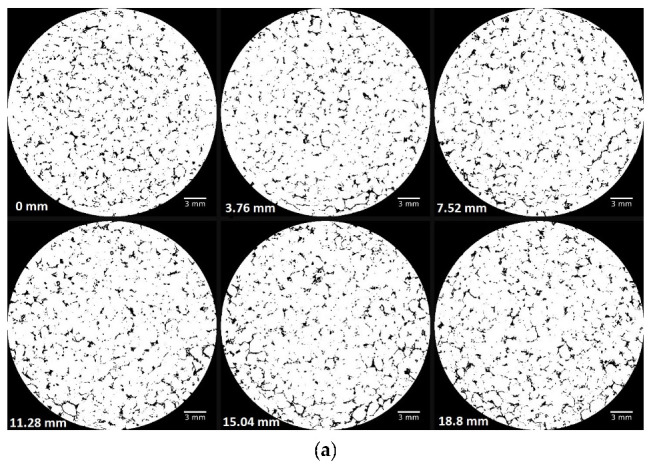

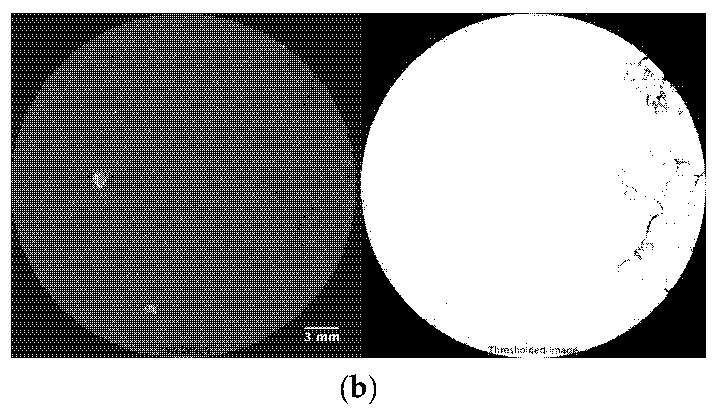
Image analysis using ImageJ software for dry granular samples after one-dimensional compression: (**a**) HN grain sample; (**b**) GLAG grain sample.

Figure 19a shows six different threshold and binarized images (final height = 18.8 mm) distributed equally throughout the entire height of the sample after compression. The analysis of these images indicates the presence of uncrushed HN grains, as well as partially crushed grains and totally crushed ones (fines). The void ratio estimated using these images, has been determined to be 8%, and was compared to the theoretical one represented by the blue plot in Figure 14. The blue curve (dry HN sample) shows that the final void ratio is almost 8.2%. Figure 19b depicts the raw and the threshold images of the compressed GLAG grain sample. Based on the resolution used (~20 µm/pixels), the analysis of the image indicates the propagation of cracks at the border of the sample and no voids were detected. The outcome of the images can be confirmed by the red plot in Figure 14, where the void ratio at the end of the test is shown to be almost zero. The finding that corresponds to the change in material behavior, before and after compression using X-ray tomography, shows high consistency with the ones coming from the data processing represented by the variation of the void ratio with respect to logarithmic vertical stress curves.

## 6. Results and Discussions

In the present work, the mechanical behavior of granular media, when subjected to an axial stress of up to 500 MPa, is represented by $e-log\sigma_{v}$ plots. These plots show three important regimes. The first regime is the quasi-elastic behavior, where the grain distribution starts to change without any crushing (several MPa), i.e., slight decrease in the void ratio, until reaching the point of maximum curvature. The yielding point defined by the second regime indicates the beginning of particle crushing, and shows a dependency on the material type, moisture content, and grains size. Afterwards, a significant decrease in the void ratio while increasing the vertical stress indicates the beginning of grain breakage and thus the appearance of a third regime. After performing 500 MPa of axial stress, we can conclude that, above 100 MPa, the slop of $e-log\sigma_{v}$ starts to become gentler, which indicates that the grains are compacted, and the sample starts to harden. Furthermore, the difference in sample’s height (Δh) before and after oedometric tests points out that calcareous sand is more compressible than siliceous sand. In other words, calcareous material undergoes higher settlement than siliceous one. In addition, the initial height of the sample reveals a considerable impact on the interaction of $e-log\sigma_{v}$ plots. Moreover, the grain breakage is enhanced by the presence of water i.e., the higher the saturation ration the higher the particle breakage. For the same material, the crushing depends significantly on the grain size; in other words, the bigger the grain the lower the degree of damage. Adding to that, the compressibility index, i.e., the slope of the third regime of $e-log\sigma_{v}$ curve, depends on the evolution of fractal geometry; the slope of the graphs shows that $C_{C}(Hustun)>C_{C}(Bocahut)$. Finally, after compression, not all voids are detectable; there are some of them between the crushed grains.

## 7. Summary and Conclusions

The behavior of sand material when subjected to uniaxial confined compression of 500 MPa was studied by means of quasi-oedometric tests. The investigation was conducted to evaluate the impact of particle size distribution, grain nature, and water saturation degree on the mechanical response of the siliceous and calcareous material. To achieve our purpose in reaching very high stress levels and, therefore, a significant settlement, a modified set up of a quasi-oedometric test apparatus was developed. In addition, 2D axisymmetric numerical simulations were applied to estimate the radial stress from the radial gages reading. Image analysis using X-ray tomography was done on dry calcareous and siliceous sand samples, revealing a significant consistency with the plots. Eight compression tests were performed and analyzed with the proposed methodology of processing. They reveal that a high degree of compaction strain (50%) can be achieved with the present test design. Stresses up to 500 MPa, confinement pressures up to 170 MPa and a strain rate of 5µm/s portray a significant variation in the state of granular material. However, this variation depends strongly on the nature of media, the grain size and the moisture content.

## Figures and Tables

**Table 1:** Sand description: shape, mean diameter, and particle density.

Material	Grain Shape	D50 (mm)	$\rho_{s}$ $\left(\;g/cm^{3}\right)$
Hostun sand HN31	Sub-angular	0.328	2.65
Hostun sand HN1.25/1.6	Sub-rounded	1.415	2.65
Glageon sand GLAG1.25/1.6	Angular	1.425	2.54

**Table 2:** Properties of quasi-oedometric tests.

Test Name	$W_{c}\;(\%)$	$D_{50}\;(\mathrm{mm})$	Dry Mass (g)	$\mathrm{Specimen}\;\mathrm{Volume}\;(cm^{3})$	$\mathrm{Dry}\;\mathrm{Density}\;(g/cm^{3})$	$\mathrm{Bulk}\;\mathrm{Density}\;(g/cm^{3})$	Void Ratio e	Porosity n	$S_{r}\;(\%)$
HN-S-0	0	0.328	31.9	21.81	1.461	1.461	0.813	45	0.00
HN-L-0	0	1.415	34.3	21.79	1.574	1.574	0.684	41	0.00
GLAG-L-0#1	0	1.425	32.8	21.71	1.508	1.508	0.757	43	0.00
GLAG-L-0#2	0	1.425	32.3	21.69	1.489	1.489	0.779	44	0.00
GLAG-L-5	5	1.425	32.8	21.76	1.506	1.585	0.759	43	18.36
HN-L-5	5	1.415	34.5	21.78	1.582	1.665	0.675	40	20.65
GLAG-L-10	10	1.425	32.6	21.73	1.501	1.667	0.766	43	38.45
HN-L-10	10	1.415	34.2	21.69	1.578	1.753	0.679	40	43.34

**Table 3:** Recapitulative table of quasi-oedometric test results.

Test Name	$W_{c}\;(\%)$	$S_{r}\;(\%)$	Mass (g)	$\Delta_{h}\;=\;h_{i}-h_{f}\;(\mathrm{mm})$	$\mathrm{Max}.\;F_{axial}\;(\mathrm{kN})$	$\mathrm{Max}.\;\sigma_{a}\;(\mathrm{MPa})$	$\mathrm{Max}.\;\varepsilon_{r}$	$\mathrm{Max}.\;\sigma_{r}\;(\mathrm{MPa})$
HN-S-0	0	0	31.87	30.9 − 19.4 = 11.5	340	507	6.9 × 10^−4^	144
HN-L-0	0	0	34.29	30.9 – 20.0 = 10.9	342	510	7.1 × 10^−4^	141
GLAG-L-0#1	0	0	32.74	30.7 − 16.3 = 14.4	341	508	6.0 × 10^−4^	145
GLAG-L-0#2	0	0	32.31	30.7 − 16.6 = 14.1	341	509	5.9 × 10^−4^	148
GLAG-L-5	5	18.4	34.49	30.8 − 15.0 = 15.8	340	507	4.8 × 10^−4^	136
HN-L-5	5	20.6	36.26	30.8 − 19.6 = 11.2	340	506	6.5 × 10^−4^	133
GLAG-L-10	10	38.4	36.24	30.8 − 15.5 = 15.3	340	507	6.2 × 10^−4^	167
HN-L-10	10	43.3	38.02	30.7 − 18.8 = 11.9	340	506	6.1 × 10^−4^	132

## Data Availability

We choose to exclude this statement since this study did not report any data.
